# The effect of interventions aiming to optimise the prescription of antibiotics in dental care—A systematic review

**DOI:** 10.1371/journal.pone.0188061

**Published:** 2017-11-14

**Authors:** Christin Löffler, Femke Böhmer

**Affiliations:** Institute of General Practice, Rostock University Medical Center, Rostock, Germany; The Ohio State University, UNITED STATES

## Abstract

**Background:**

Abundant evidence in dentistry suggests that antibiotics are prescribed despite the existence of guidelines aiming to reduce the development of antibiotic resistance. This review investigated (1) which type of interventions aiming to optimise prescription of antibiotics exist in dentistry, (2) the effect of these interventions and (3) the specific strengths and limitations of the studies reporting on these interventions.

**Method:**

Literature search was based on Medline, Embase, Global Health, Cochrane CENTRAL, ClinicalTrials.gov and Current Controlled Trials. Studies with one of the two primary outcomes were included: (1) The number of antibiotics prescribed and/or (2) the accuracy of the prescription, commonly measured as a percentage of adherence to local clinical guidelines.

**Results:**

Nine studies met these inclusion criteria. Five studies reported on the prescription of antibiotics in primary dental care and four studies focused on outpatient dental care. Interventions used in primary dental care included a combination of audit, feedback, education, local consensus, dissemination of guidelines and/or academic detailing. Trials in the outpatient setting made use of expert panel discussions, educational feedback on previous acts of prescribing, the dissemination of guidelines and the establishment of internal guidelines. All studies successfully reduced the number of antibiotics prescribed and/or increased the accuracy of the prescription. However, most studies were confounded by a high risk of selection bias, selective outcome reporting and high variance across study groups. In particular, information relating to study design and methodology was insufficient. Only three studies related the prescriptions to the number of patients treated with antibiotics.

**Conclusions:**

This systematic review was able to offer conclusions which took the limitations of the investigated studies into account. Unfortunately, few studies could be included and many of these studies were confounded by a low quality of scientific reporting and lack of information regarding study methodology. High-quality research with objective and standardised outcome reporting, longer periods of follow-up, rigorous methodology and adequate standard of study reporting is urgently needed.

## Introduction

Today, appropriate prescription of antibiotics is a challenge in most health care systems.[1–6] Inadequate prescribing accelerates the process for antibiotic resistance development and has serious consequences for worldwide health care. Presently, antibiotic resistance is one of the biggest threats to global health and results in less effective therapies for a growing number of infections, longer hospital stays, higher medical costs and increased mortality.[7]

Supranational bodies are starting to address this challenge.[8] Nonetheless, coordinated action at the national and international level is late and still insufficient.[9]

Although the inappropriate use of antibiotics rose across Europe between 1997 and 2009, there has been no significant increase from 2011 to 2015.[10, 11] With 7–10% of all antibiotics used in outpatient care, dentistry accounted for a comparatively high amount of these prescriptions.[12–14] In contrast to the increase in the prescription of antibiotics found during the 1990s, there now is some evidence suggesting that prescribing rates are reaching a plateau in dentistry.[15]

However, we found abundant evidence, particularly from the UK, that a high number of antibiotics were provided despite being incompatible with guidelines in dentistry.[16–18] Most commonly, antibiotics were prescribed for irreversible pulpitis[19], chronic periodontitis[20], acute dental problems[21], removal of third molars[22] or as prophylactic treatment against implant failure.[23] There is ongoing controversy whether antibiotics are necessary in most of these conditions and a lot of studies confirmed that dentists around the world prescribe antibiotics contrary to local guidelines.[15, 24–40]

Underlying reasons for the misuse of antibiotics included dentists’ desire to avoid clinical complications, the fear to lose patients and perceived patient pressure.[41, 42] Furthermore, dentists were inclined to put their professional experience before guidelines.[41] In Wales, for instance, merely 19% of all antibiotic prescriptions by General Dental Practitioners (GDP) were compatible with the guidelines.[25] Several studies confirmed the urgent need for dental undergraduate and postgraduate education in the prescription of antibiotics.[16, 17, 42–44]

For many years, the prophylactic prescription of antibiotics has been important for the prevention of infective endocarditis among at-risk patients undergoing invasive dental treatment.[45–48] Recently, however, a number of clinical guidelines restricted clinical conditions that require such prophylaxis.[49] [50] [51] Currently, there is vigorous debate whether this shift in guidelines might lead to an increased incidence of endocarditis.[52, 53]

Surprisingly, antibiotic misuse is rarely addressed publically or scientifically in dentistry, which is in contrast to the practice found in general medicine. So far, only a small number of interventional trials have been conducted in this field. Most of these trials made use of cognitive elements such as clinical audits, educational outreach visits or feedback. However, there was no systematic evidence, whether these interventions were able to produce sustained changes in the prescription of antibiotics.

The present systematic review aimed at investigating whether the interventions were associated with changes in the prescription (reduction of the number of prescriptions or changes in accuracy) in general dental care and in specialized dental care. In particular, this review addressed three objectives: Firstly, which types of interventions aiming to optimise the prescription of antibiotics were reported in dentistry? Secondly, what was the effect of these types of interventions and which types were most effective? And last but not least, what were the specific strengths and limitations of the studies included and what was their impact on data validity aspects?

## Material and methods

Literature search methodology, data extraction, synthesis and reporting were based on the Cochrane handbook for systematic reviews of interventions[54] and the PRISMA statement for preferred reporting items for systematic reviews and meta-analyses.[55]

### Literature search and selection criteria

The literature search included bio-medical academic peer-reviewed original research from Medline, Embase, Global Health and Cochrane CENTRAL. Additionally, the clinical trials registries ‘ClinicalTrials.gov’ and ‘Current Controlled Trials’ were searched in order to identify ongoing trials whose findings were not yet published and to compare published studies with protocols.

The search included studies investigating the effect of all types of interventions aiming to optimise acts of prescribing antibiotics in dentistry, such as clinical audits, educational outreach visits, feedback, patient education and communication training. Studies investigating effects in primary dental care and specialised dental care were included. We aimed to investigate two primary outcomes: (1) The number of prescriptions for antibiotics and/or (2) the accuracy of the prescription, commonly measured as a percentage of adherence to local clinical guidelines.

Since only few studies have addressed the optimisation aiming to impact on acts of prescribing antibiotics in dentistry so far, all quantitative studies were included in this review. This was necessary, as this review could not be limited to randomised controlled trials. Databases were searched for English and German entries dated January 1960 and later with no restrictions on their geographical focus.

Search terms included synonyms as well as major subject headings and subheadings that had been adjusted to the database. Search concepts were based on "antibiotic prescribing", "dentistry" and "intervention". See Table 1 for full search terms by database. To manage literature entries the software program EndNote was used.

**Table 1:** Search terms by database.

Database	Search term
Medline (Ovid)	1. (Antibiotic* OR anti-bacterial* OR prophylactic*) ADJ3 (prescrib* OR prescription OR agent* OR therapy OR therapeutic*) 2. exp Anti-Bacterial Agents/ 3. 1 OR 2 4. (dentist* OR dental care OR dentistry OR dental surgeon* OR dental practitioner* OR general dental practice* OR general dental practitioner*) 5. exp Dentistry/ 6. 4 OR 5 7. (intervention* OR dental audit* OR clinical audit* OR educational outreach visit* OR peer visit* OR feedback OR guideline* OR communication* OR intervention* OR stud* OR trial*) 8. exp clinical study/ 9. 7 OR 8 10. 3 AND 6 AND 9 11. limit 10 to (English or German) 12. limit 11 to humans Note: No MeSH "intervention" or "intervention study" in Medline.
Embase (Ovid)	1. (Antibiotic* OR anti-bacterial* OR prophylactic*) ADJ3 (prescrib* OR prescription OR agent* OR therapy OR therapeutic*) 2. exp antiinfective agent/ 3. 1 OR 2 4. (dentist* OR dental care OR dentistry OR dental surgeon* OR dental practitioner* OR general dental practice* OR general dental practitioner*) 5. exp dentistry/ 6. 4 OR 5 7. (intervention* OR dental audit* OR clinical audit* OR educational outreach visit* OR peer visit* OR feedback OR guideline* OR communication* OR intervention* OR stud* OR trial*) 8. exp intervention study/ 9. 7 OR 8 10. 3 AND 6 AND 9 11. limit 10 to (English or German) 12. limit 11 to humans
Global Health (Ovid)	1. (Antibiotic* OR anti-bacterial* OR prophylactic*) ADJ3 (prescrib* OR prescription OR agent* OR therapy OR therapeutic*) 2. exp antiinfective agent/ OR exp antibiotics/ 3. 1 OR 2 4. (dentist* OR dental care OR dentistry OR dental surgeon* OR dental practitioner* OR general dental practice* OR general dental practitioner*) 5. exp dentistry/ 6. 4 OR 5 7. (intervention* OR dental audit* OR clinical audit* OR educational outreach visit* OR peer visit* OR feedback OR guideline* OR communication* OR intervention* OR stud* OR trial*) 8. exp intervention/ OR exp feasibility studies/ OR exp implementation of research/ OR exp medical research/ OR clinical trials/ 9. 7 OR 8 10. 3 AND 6 AND 9 11. limit 10 to (English or German) (Note: Not possible to limit to humans)
Cochrane CENTRAL	1. (Antibiotic* OR anti-bacterial* OR prophylactic*) NEAR/3 (prescrib* OR prescription OR agent* OR therapy OR therapeutic*) 2. exp antibacterial agents 3. 1 OR 2 4. (dentist* OR dental care OR dentistry OR dental surgeon* OR dental practitioner* OR general dental practice* OR general dental practitioner*) 5. exp dentistry 6. 4 OR 5
ClinicalTrials.gov	(antibiotic OR anti-infective OR antibacterial) AND (dentistry OR dental)
Current Controlled Trials	(antibiotic OR anti-infective OR antibacterial) AND (dentistry OR dental)

### Data extraction and synthesis

Both reviewers independently determined the eligibility of studies, assessed the methodology of the included studies and extracted the data. Studies that met the inclusion criteria were included in the review. A piloted data extraction sheet formed the basis for data extraction from these studies. Among other items, this sheet included information about study objective, design, participants, intervention(s), outcome(s) and result(s). Authors were contacted in order to resolve open questions. A narrative synthesis is provided within this review which summarises the study results with respect to their objectives, settings, interventions and effects. Particular emphasis is given to the guidelines used to determine the accuracy of the prescription for an antibiotic. A combination of the Cochrane Collaboration's tool for assessing the risk of bias[54] and the STROBE statement for reporting of observational studies in epidemiology[56] was employed to assess the risk of bias in each study. A risk of bias sheet including eleven domains was developed and piloted. Information on each domain was extracted from the publications, obtained from personal correspondence or was based on our judgement. The narrative synthesis of all studies is presented alongside the summary table and figure.

### Registration

This review was registered at the International Prospective Register of Systematic Reviews (PROSPERO) on August 2nd, 2016 under the registration number CRD42016043154.

## Results

### Study selection

**Fig 1:**
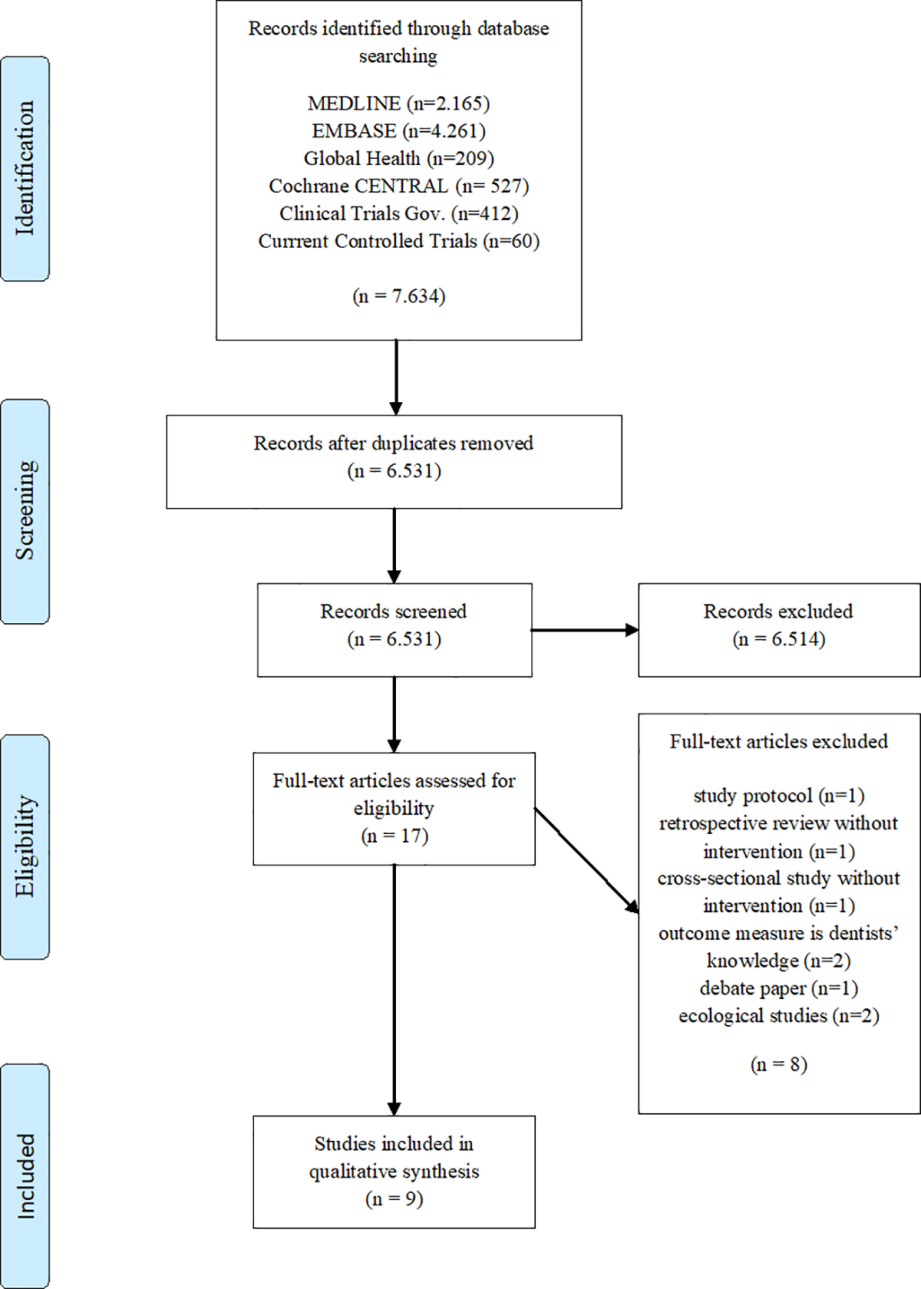
Study selection process. Note: PRISMA flow chart based on: Moher D, Liberati A, Tetzlaff J, Altman DG, The PRISMA Group (2009). Preferred Reporting Items for Systematic Reviews and Meta-Analyses: The PRISMA Statement. PLoS Med 6(7): e1000097.

Databases were searched on the 20^th^ of June in 2016 and a sum of 7.634 publications was found. 6.531 entries remained in the Endnote database after removal of duplicate records. A total of 17 potentially relevant publications were retrieved for full paper review. Reasons for excluding papers from the review comprised a lack of intervention[57, 58], wrong outcome measures[59, 60] and wrong study design (ecological studies)[52, 53]. One debate paper[61], early-stage trials and those without published findings were also excluded.[42, 62] Findings of one of the later trials were published during the review process, thus this publication could be included in our review.[63] Finally, nine studies met the inclusion criteria (Fig 1).

### Study objectives and settings

All studies had been conducted within the UK, apart from one study which had been administered in Nepal.[64] Five studies aimed to optimise the prescription of antibiotics in primary dental care[63, 65–68], while four studies focused on outpatient dental care. Within those studies assessing practice in primary dental care, four trials included all medical conditions[63, 65, 66, 68, 69], whereas one study exclusively investigated acute dental pain.[67] Among the studies that were conducted in outpatient care, two studies focused on all conditions.[64, 69] Chopra et al. conducted their study in a department mainly treating patients with acute dental pain and infections.[70] Thomas and Hill included patients undergoing third molar surgery.[71] All studies aimed at reducing the inappropriate prescription of antibiotics in dental care by decreasing the number of antibiotics per prescription and/or by increasing the level of compliance with local or national guidelines. For example, these guidelines involved considerations about the clinical indication for a prescription of an antibiotic, the recommended antimicrobial agent, dose, frequency and duration of intake (Table 2).

**Table 2:** Characteristics of included studies: Country, setting, clinical condition and objective.

Authors and year	Country	Setting	Clinical condition	Objective
Elouafkaoui et al. 2016[63]	Scotland, UK	NHS General dental practices	All conditions	"To compare the impact of individualized audit and feedback interventions on dentists' antibiotic prescribing rates."[63]
Chate et al. 2006[65]	Eastern England, UK	General dental practices	All conditions	"To reduce the number of antibiotics inappropriately prescribed by general dental practitioners, and to increase overall prescription accuracy."[65]
Palmer et al. 2001[66]	North West of England, UK	General dental practices	All conditions	"To investigate whether clinical audit can improve general dental practitioners' prescribing of antibiotics."[66]
Seager et al. 2006[67]	Wales, UK	General dental practices	Acute dental pain	"To assess the effect of educational outreach visits on antibiotic prescribing for acute dental pain in primary care."[67]
Steed and Gibson 1997[68]	Scotland, UK	General dental practices	All conditions	"Investigated the rationale of general dental practitioners for antibiotic prescribing and the compliance and understanding of patients in the use of antibiotics as part of their dental care. Following the model for clinical audit and reviewing antibiotic prescribing thereafter."[68]
Zahabiyoun et al. 2015[69]	North East of England, UK	Outpatient clinics	All conditions	"To determine whether the prescriptions comply with the recommended guidelines and whether clinical audit can alter the prescribing practices of dentists leading to better use of antibiotics in the dental service."[69]
Chopra et al. 2014[70]	London, UK	Outpatient clinic	Mainly acute dental pain and infection	"To audit how appropriately antimicrobials were prescribed in the oral surgery acute dental department of Guy's Hospital in London, when compared to the standards set within the Faculty of General Dental Practice (UK) and Scottish Dental Clinical Effectiveness guidelines on antimicrobial prescribing in dentistry."[70]
Raunair et al. 2012[64]	Nepal	Outpatient clinic	All conditions	"To measure the impact of educational feedback intervention on the prescribing behavior of dental surgeons."[64]
Thomas and Hill 1997[71]	UK	Outpatient clinic	Third molar surgery	"To rationalize antibiotic prescribing in third molar surgery to a defined standard and to re-audit prescribing patterns to determine whether the rationalization of antibiotic prescription could be maintained without affecting surgical outcome."[71]

### Study designs

Of the nine studies included in this review, two were randomised controlled trials (RCT): In a three-arm RCT, Seager et al. compared two interventions with usual care. Prior to randomisation, the participating practices were stratified by their previous level of prescribing antibiotics.[67] Elouafkaoui et al. compared the impact of individualised audit and feedback interventions on the rate of prescribing antibiotics by conducting a three-arm partial factorial cluster RCT. They compared practices randomised to individualised graphical audit and feedback with and without a written behaviour change message (arm 1 and 2, respectively) against care as usual (arm 3). Among both intervention arms, participating practices were randomly allocated to receive audit and feedback: (i) with or without a health board comparator and (ii) at zero, six months or at zero, six and nine months into the study.[63] All other studies[64–66, 68–71] made use of uncontrolled pre-post designs, while most of them compared the practice of prescribing antibiotics over a comparatively short period before and after the intervention.[65, 66, 71] Other studies included a fixed number of patients or prescriptions of antibiotics.[64, 69, 70] Only Raunair et al. compared the practice of prescribing antibiotics between more than two time points: before intervention and at one, three and six months after intervention.[64] In all studies the follow-up period ranged between six weeks and twelve months. Four trials were comparatively large and included up to some thousand prescriptions or patients.[63–67] The other studies included between 55 and 320 prescriptions.[68–71] Some studies quantitatively evaluated prescriptions for antibiotics[64, 69, 70], whereas others sampled participating dentists (Table 3).[65–67]

**Table 3:** Characteristics of included studies: Study design, intervention, time periods, sample size and outcome measures.

Authors and year	Study design	Intervention	Baseline Period	Intervention Period	Time between Intervention and Follow-up	**Follow-up Period**	**Sample Size**	**Outcome measures**
Elouafkaoui et al. 2016	Partial factorial cluster RCT	Audit and feedback with and without written behaviour change message (i) with and without a health board comparator and (ii) at 0 and 6 or at 0, 6 and 9 months)	12 months (data provided by the Management Information and Data Accounting System database)	At 0, 6 or 0, 6 and 9 months, depending on study group	Immediate	12 months (**from May 2013 to April 2014)**	795 practices with 2,566 GDPs	**Total number of antibiotic items dispensed per 100 NHS treatment claims,** total number of Amoxicillin 3g dispensed per 100 NHS treatment claims, total number of broad spectrum antibiotics dispensed per 100 NHS treatment claims, prescription rates of DDD
Chate et al. 2006	Pre-post design	Audit (feedback) and education, guidelines and local consensus	6 weeks	No information	Immediate	6 weeks	212 GDPs (4.616 prescriptions for antibiotics)	**Prescriptions for antibiotics**, prescription for antimicrobials, clinical and medical conditions, regimen of antibiotic: dose, frequency and duration of use, **compliance with guidelines**
Palmer et al. 2001	Pre-post design	Audit (feedback) and education, guidelines and local consensus	6 weeks	No information	Immediate	6 weeks	175 GDPs (3.646 prescriptions for antibiotics)	**Prescriptions for antibiotics**, prescriptions for antimicrobials, reasons for therapeutic prescriptions, reasons for prophylactic prescriptions, **compliance with guidelines**, regimen of antibiotic: correct dose, frequency and duration of Amoxicillin / Metronidazole when prescribed
Seager et al. 2006	RCT	Group 1: Provision of educational material Group 2: Provision of educational material and academic detailing visit by pharmacist	No baseline period	No information	Immediate	3 months ^(a)^	1.497 patients aged 16+ with acute dental pain from 70 GDPs (416 antibiotic prescriptions)	**Number of prescriptions for antibiotics** issued to patients presenting with acute dental pain, **number of inappropriate prescriptions**
Steed and Gibson 1997	Pre-post design	Consensus based design of intervention material e.g. guideline checklist, aide-mémoire	4 months	4 months	Immediate	4 months	320 prescriptions for antibiotics from 15 GDPs at baseline	**Number of prescriptions** issued before and after intervention by practice
Zahabiyoun et al. 2015	Pre-post design	Expert panel discussion and dissemination of guidelines	No baseline period (retrospective record review)	No information	Immediate	No information	55 prescriptions for antibiotics	**Number of prescriptions in accordance with recommended standards**, number of prescriptions in accordance with recommended standards for a) Metronidazole and b) Amoxicillin
Chopra et al. 2014	Pre-post design	Audit and education, dissemination of guidelines	No information	2 months	Immediate	No information	120 patients with prescriptions for antimicrobials (60 pre and 60 post)	**Compliance with guidelines**, recording of diagnosis, incorrect dose of Amoxicillin when prescribed
Raunair et al. 2012	Pre-post design	Educational feedback on prescribing behaviour	No information	No information	Immediate	At 1, 3 and 6 months after intervention	1.200 outpatient prescriptions—300 per point of measurement (500 prescriptions for antibiotics)	Mean number of drugs per prescription, total number of prescriptions with antimicrobial agents, **total number of antimicrobial agents**, mean number of antimicrobial agents per prescription, other drugs on prescription, most commonly prescribed drugs
Thomas and Hill 1997	Pre-post design	Establishing an internal guideline	1 month	No information	No information	1 month (one year after baseline)	132 patients undergoing general anaesthesia for the removal of third molar teeth (132 prescriptions for antibiotics)	**Preoperative prescriptions for antibiotics**, **postoperative prescriptions by antibiotic substance**, presence of postoperative infection at 1 week, number of postoperative visits, patient attendance at practitioners outside the hospital

Note

(a) Information based on trial registration at Current Controlled Trials ISRCTN51223556; information not provided by the publication. RCT = randomised controlled trial; GDP = general dental practitioner; NHS = National Health Service; DDD = defined daily dose.

### Description of interventions

Audit, feedback, education, local consensus and the dissemination of guidelines were the elements most often used and combined in the studies.[63, 65–68, 70] Elouafkaoui et al. provided graphical individualised audit and feedback, written behaviour change messages and a health board comparator.[63] Seager et al. compared the effect of an academic detailing visit by a trained pharmacist and the dissemination of guidelines to care as usual and to the dissemination of guidelines alone.[67] Steed and Gibson designed intervention material such as checklists and an aide-mémoire that were based on previous consensus.[68] Zahabiyoun et al. investigated the effect of an expert discussion panel.[69] Thomas and Hill established internal guidelines.[71] And Raunair and colleagues provided educational feedback on previous prescribing behaviour (Table 3).[64]

### Reporting of outcomes

Three studies collected outcome data on the number of the prescribed antibiotics and the act of prescribing antibiotics as a measure of adherence to local or national guidelines.[65–67] Four studies provided data on the number of antibiotics that had been prescribed [63, 64, 68, 71] and two studies investigated compliance with guidelines.[69, 70]

#### Number of prescribed antibiotics

Comparing the number of prescribed antibiotics across studies was challenging as only three studies related the prescriptions to the number of patients treated.[63, 67, 71] The other studies do not report this relationship and implicitly assumed that the number of patients treated by dentists would not change over time.[64–66, 68] Most studies also did not report on confidence intervals, p-values and standard deviations. Only Elouafkaoui et al. and Raunair et al. provided more sophisticated outcome measures. These included the number of antibiotic items dispensed per 100 treatment claims, the number of prescriptions for antimicrobial agents, the number of antimicrobial agents and average of antimicrobial agents per prescription.[63, 64]

**Fig 2:**
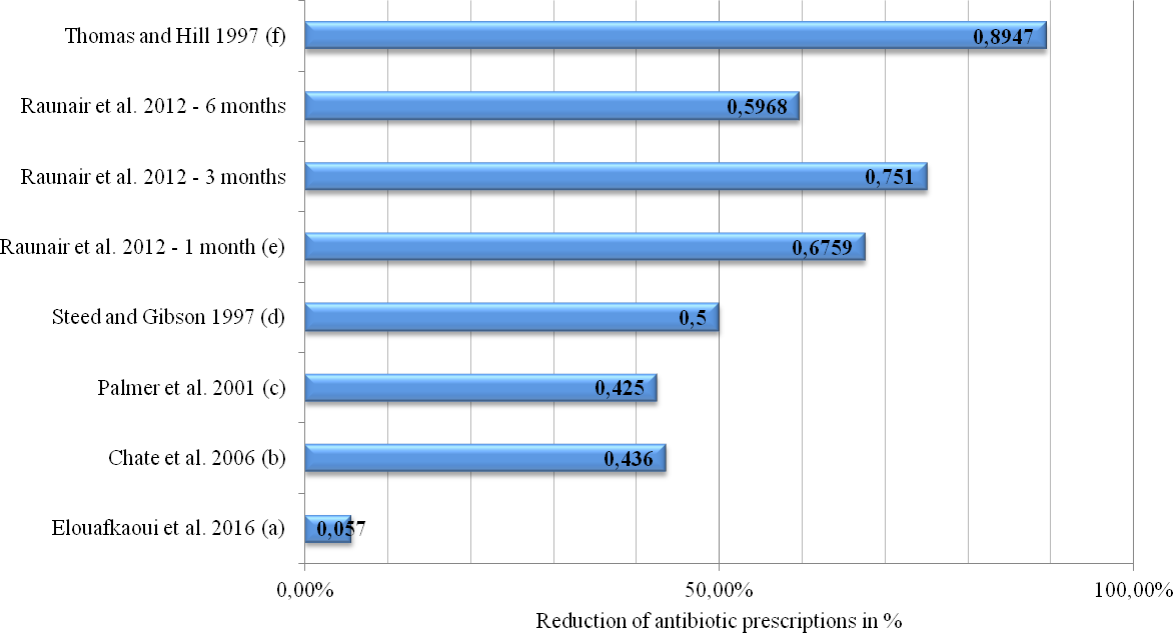
Decrease in the number of prescriptions for antibiotics in percentage, by studies assessing this outcome parameter. Note: Chopra et al. (2014) and Zahabiyoun et al. (2015) do not report that outcome measure. Instead of reporting figures, odds ratios are reported in Seager et al. (2006). (a) Reduction of 1.0 antibiotic items per 100 NHS treatment claims in the intervention group compared to 0.4 antibiotic items in the control group. (b) Reduction from 2,951 prescriptions for antibiotics before the audit to 1,665 prescriptions after the audit. (c) Reduction from 2,316 prescriptions for antibiotics before the audit to 1,330 prescriptions after the audit. (d) The number of prescriptions was not stated in the paper, but the authors report an overall reduction of ~50%. (e) Reduction of the total number of prescriptions for antimicrobial agents from 253 prescriptions among 300 patients at baseline to 82 prescriptions among 300 patients at one month after the intervention. Three months after the intervention these prescriptions were at 63 among 300 patients and at 102 prescriptions among 300 patients six months after the intervention. (f) Reduction of preoperative prescriptions for antibiotics from 15 prescriptions among 80 patients before the audit to one prescription among 52 patients after the audit. A postoperative reduction of prescriptions for antibiotics was not intended.

Bearing these limitations in mind, within those studies following the pre-post design, interventions were associated with a reduction of prescriptions for antibiotics ranging between 42.5% and 89.5%. In the large studies by Palmer et al.[66] and Chate et al.[65] reductions reached 42.5% and 43.6%, respectively. Smaller trials, mostly conducted in outpatient departments, showed higher levels.[64, 71] Raunair et al. witnessed a reduction of prescriptions for antibiotics one month after intervention by almost 68% and this reduction increased to 75% at three months after intervention. However, at six months after intervention the reduction regressed to 60%.[64] All of these reductions were statistically significant. The other studies did not report p-values or significance levels (Fig 2). In their three-arm RCT, Seager et al. compared the odds of dentists prescribing antibiotics among different intervention groups. Compared to the control group, patients from the group of dentists accepting guidelines had 17% lower odds of receiving antibiotics (95% Confidence Interval (CI): 0.55; 1.21). Relative to the control group, patients of dentists receiving an academic detailing visit by a trained pharmacist had 37% lower odds that they would be given a prescription for antibiotics (95% CI: 0.41; 0.95).[67] The Scottish cluster RCT testing an audit and feedback intervention was able to reduce the number of prescriptions for antibiotics. Specifically, 8.5 prescription items per 100 NHS treatment claims were reduced to 7.5 antibiotic items within the intervention groups, compared to only 0.4 items (from 8.3 to 7.9 items) in the control group. The overall adjusted effect size of 0.47 (95% CI: 0.01; 0.85) was significant (p = 0.01). Relative to the control group this is a reduction of 5.7% (95% CI: -1.1%; -10.2%) in the rate of prescribing antibiotics (p = 0.01).[63]

Interventions most strongly associated with reducing the number of prescriptions for antibiotics include the establishment of internal guidelines[71] and educational feedback on previous acts of prescribing antibiotics.[64] Both interventions were tested in outpatient departments involving comparatively few patients. The combination of audit, education, local consensus and dissemination of guidelines showed less pronounced but still high levels of reductions.[65, 66] These complex interventions were settled in primary dental care and included a higher number of patients. The audit and feedback intervention involving all NHS general dental practices in Scotland showed the weakest reduction in the number of prescriptions.[63]

#### Adherence to guidelines

As far as adherence to guidelines is concerned, studies vary by the type of guidelines used. Whereas four trials relied on national guidelines[65, 66, 69, 70], three studies established local guidelines.[67, 68, 71] Raunair et al. and Elouafkaoui et al. did not investigate compliance to guidelines (Table 4).[63, 64]

**Table 4:** Characteristics of the studies and their guidelines.

Authors and year	Guideline(s)
Elouafkaoui et al. 2016	n/a
Chate et al. 2006	Guidelines of the Faculty of General Dental Practitioners, Royal College of Surgeons of England published in 2000[72] (adults), British National Formulary (children).
Palmer et al. 2001	Guidelines of the Faculty of General Dental Practitioners, Royal College of Surgeons of England published in 2000.[72]
Seager et al. 2006	Establishment of local guideline: in consultation with five GDPs and three general medical practitioners.[67]
Steed and Gibson 1997	Establishment of local guideline: consensus based on current practice and patient compliance.[68]
Zahabiyoun et al. 2015	Faculty of General Dental Practice (UK) guidelines on antimicrobial prescribing for general dental practitioners published in 2012.[73]
Chopra et al. 2014	Faculty of General Dental Practice (UK) guidelines on antimicrobial prescribing for general dental practitioners published in 2012[73]; dental clinical guidance from the Scottish Dental Clinical Effectiveness Programme Drug Prescribing in Dentistry published in 2011.[74]
Raunair et al. 2012	n/a
Thomas and Hill 1997	Establishment of local guideline.[71]

**Fig 3:**
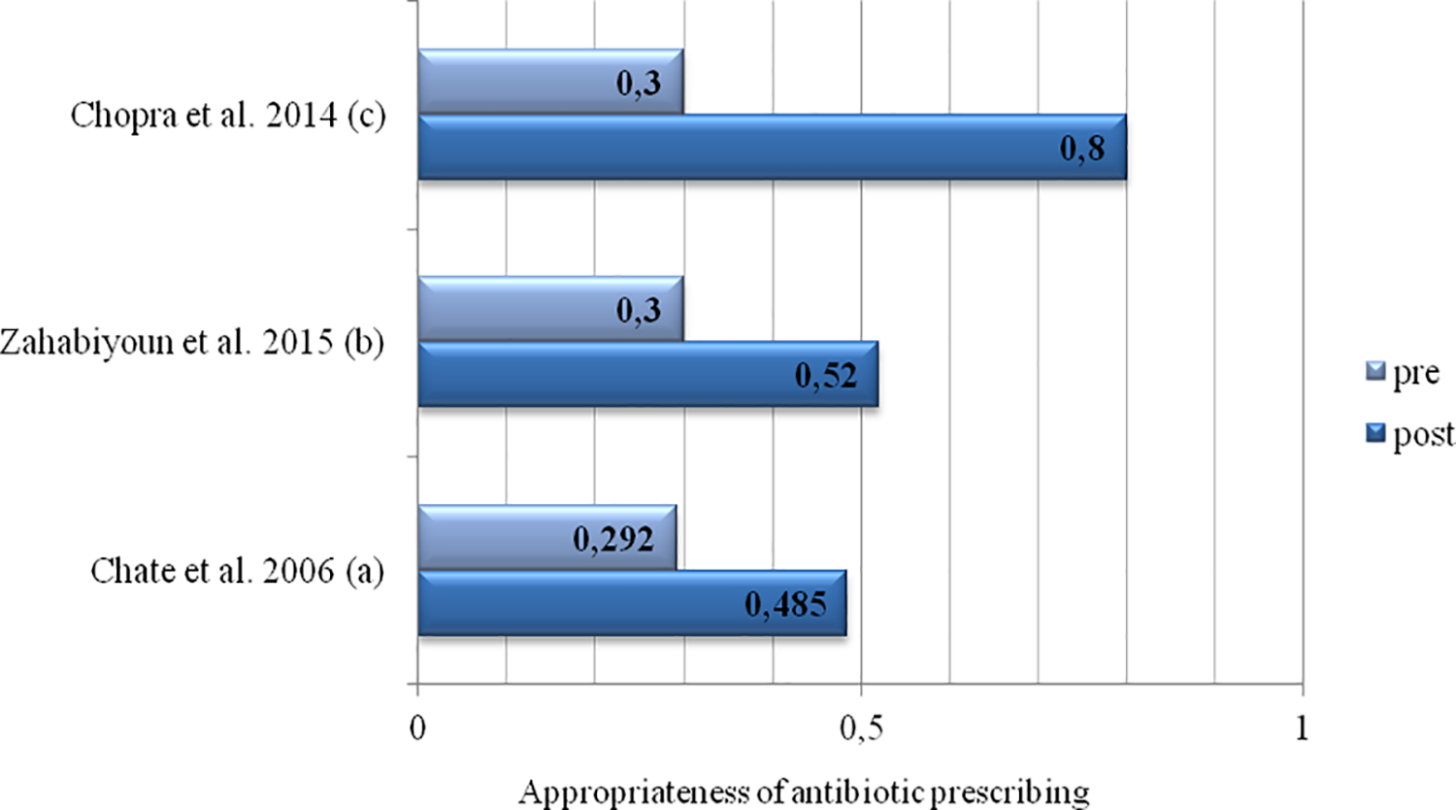
Accuracy of the prescription before (pre) and after (post) intervention, by studies assessing this outcome parameter (measured as a percentage of adherence to guidelines). Note: Palmer et al. (2001), Steed and Gibson (1997), Raunair et al. (2012) and Thomas and Hill (1997) did not report this outcome measure. Seager et al. (2006) provided information as odds ratios. (a) p<0.001. (b) p = 0.01. (c) p-values were not reported within this publication.

Although most studies made use of guidelines, merely five trials used adherence to guidelines as an outcome measure. Among the pre-post studies, three papers provided detailed information on adherence to guidelines.[65, 69, 70] Two of these studies reported significant reductions of the inappropriate prescription of antibiotics of about 50% before intervention to roughly 30% after the intervention.[65, 69] Chopra et al. observed levels of inappropriate prescribing in 80% before the intervention. Within this study these levels were reduced to 30%, however, p-values were not reported.[70] Palmer et al. also reported a significant increase in the number of appropriate prescriptions (p<0.05), but did not provide any other statistics (Fig 3).[66] Based on data from their RCT, Seager et al. estimated the odds of patients inappropriately receiving prescriptions for antibiotics. Compared to the control group, patients of dentists who received guidelines had 18% lower odds of inappropriate prescriptions (95% CI: 0.53; 1.29), while patients of dentists who were visited by a trained pharmacist had 67% lower odds (95% CI: 0.21; 0.54).[67]

The strongest effects were witnessed in the outpatient setting which included a comparatively low number of patients/prescriptions. Here, expert panel discussions and the dissemination of guidelines had been entered as interventions.[70] Marked reductions in the inappropriate prescription of antibiotics were also seen in the large studies within the primary dental care setting.[65, 67] These studies made use of complex interventions including elements of audit, education, local consensus, dissemination of guidelines and academic detailing.

#### Additional outcome measures

Additionally, the trials reported on clinical and medical conditions related to the prescription of the antibiotic as outcome measures.[65, 66] Furthermore, information derived from prescriptions for antimicrobials included duration, dose and frequency of intake (particularly with respect to Metronidazole and Amoxicillin).[65, 66, 69, 70] Raunair et al. also reported the average number of drugs per prescription, other drugs prescribed and the most commonly prescribed drugs.[64]

#### Adverse effects

Most studies did not investigate adverse effects owing to the reduction in prescriptions of antibiotics. By contrast, Thomas and Hill, who focused on the prescription of antibiotics for third molar surgery, reported the presence of a postoperative infection at one week, the number of postoperative visits and the attendance at practitioners outside the hospital. The study was not able to identify negative effects on these parameters.[71]

### Synthesis of results

All studies included in this review successfully reduced the number of prescription for antibiotic drugs and increased the accuracy of the prescription. Studies conducted in the outpatient setting usually included a lower number of patients and were more successful in reducing the number of prescriptions than interventions in primary dental care.[70, 71] The latter were often based on a comparatively high number of patients, reaching remarkable levels of reduction on prescriptions[65–67] and reported increased adherence to prescription guidelines for antibiotics.[65] Trials in the outpatient setting made use of expert panel discussions, educational feedback on previous acts of prescribing, the dissemination of guidelines and the establishment of internal guidelines.[64, 69–71] Interventions used in primary dental care included a combination of audit, feedback, education, local consensus, dissemination of guidelines and academic detailing.[63, 65–68]

### Risk of bias within studies

**Fig 4:**
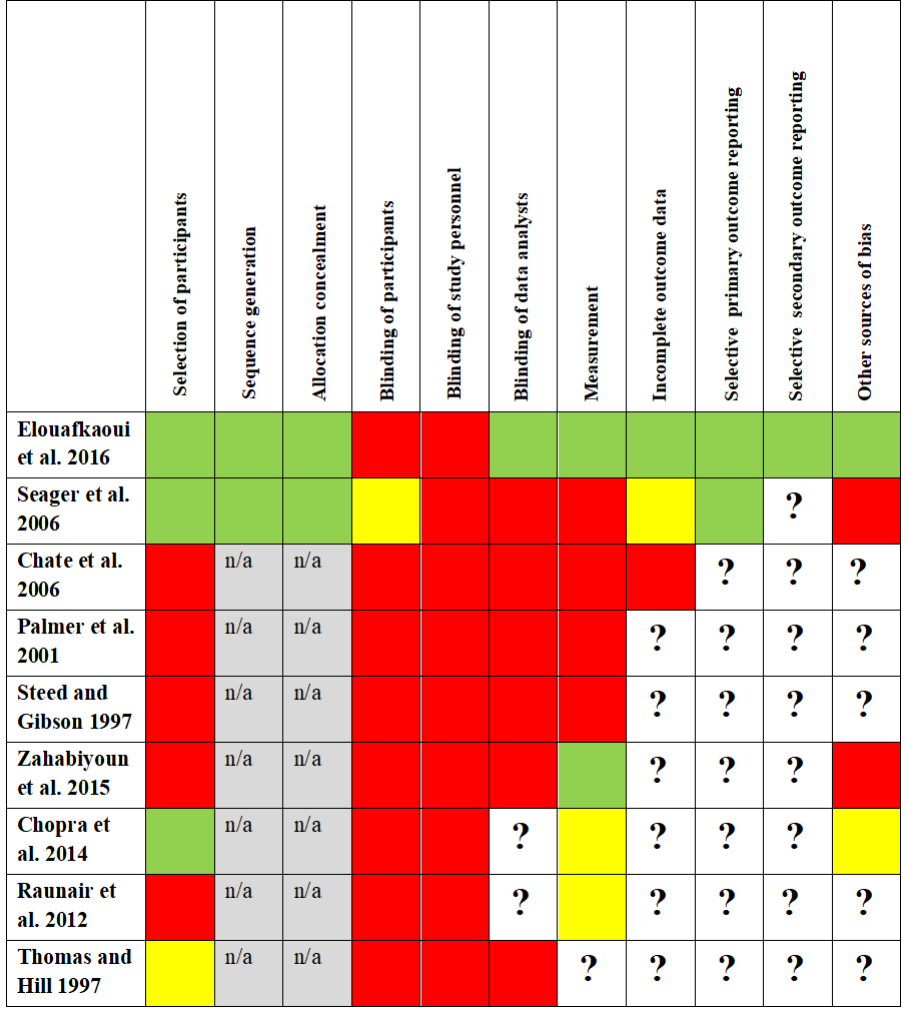
Risk of bias within included studies. Note: Low risk of bias is indicated by green colour, moderate risk of bias by yellow colour and high risk of bias by red colour. The question mark indicates an unknown risk.

A combination of the Cochrane Collaboration's tool for assessing the risk of bias[54] and the STROBE statement for reporting of observational studies in epidemiology[56] was employed in order to assess the risk of bias in RCTs and trials with a pre-post designs. Risk of bias was categorised into low, moderate, high and unknown risk. The latter category was used when publications did not provide enough information to pass judgment. Unfortunately, this was the case for a number of domains in most publications and most frequently studies from the 1990ies were affected by this lack of information (Fig 4). The highest methodological standard was witnessed in the study by Elouafkaoui and colleagues[63]: All NHS dental practices in Scotland prescribing antibiotics were included, practices were randomly allocated to groups, outcome measures were based on routinely collected NHS data and the trial statistician was blinded to allocation. These methodological features minimised the potential risk of bias. The risk of selective reporting by dentists was high in the study by Seager et al.[67] as participants were asked to complete a questionnaire every time an adult patient presented with acute dental pain. It remained unclear to what extent dental practitioners complied with these data collection procedures since compliance of dentists was not monitored and information on group differences in reporting was not included. Furthermore, there was high variance across study groups: Firstly, the attrition level differed between control, guideline and academic outreach group. Secondly, there were significant differences in the proportion of privately registered patients and in the proportion of patients with a symptom indicative of spreading infection across groups, consequently, the risk of selection bias was high. Last but not least, the trial registration provided very limited information which rendered the evaluation of outcome reporting difficult.

The studies by Palmer et al.[66] and Chate et al.[65] corresponded to each other as far as study design and procedures were concerned. In both studies, a major risk of bias arose from the fact that dentists participating in the trial were critically involved in the design and conduct of the audit: Dentists were aware of the primary outcome measure, discussed the anonymous baseline findings and set their own goals and standards in the second audit period. Additionally, the dentists were asked to use a proforma every time they prescribed an antibiotic. In consequence, there might have been a high risk of prescribing antibiotics differently than usually (Hawthorne effect) and of selective reporting. Furthermore, information upon the dropout of a dentist from the trial was not collected. The authors of these affected studies could not reconstruct if and when a dentist stopped providing information on prescribing antibiotics. This might have led to a high risk of selection bias as those dentists not complying with study goals or standards or those under time pressure might have left the audit prematurely. Quantitatively this is problematic as prescriptions for antibiotics were compared longitudinally before and after the audit. This way, dentist attrition in the second data collection period would have automatically reduced the total number of prescriptions, insinuating a successful audit.

In the study by Steed and Gibson[68] seven dental practitioners out of 15 participating practices formed the audit group, secured funding for the project, collected own prescribing data and performed the data analyses. The remaining participating dentists collaborated with the former in the same practices. Based on this strong involvement of audit participants in the audit, dentists might have influenced outcomes (sub)consciously, for example by prescribing antibiotics differently than usually (Hawthorne effect), by influencing definitions, procedures or analyses. There was also a high risk of selective reporting. Finally, it remains unclear whether there were any differences in patients and prescriptions before and after the audit, consequently, any of these variances might have distorted the comparisons.

In the study by Zahabiyoun et al.[69] the external validity might be low as the second participating clinic was chosen explicitly because of its high number of unscheduled emergency visits leading to high rates of prescriptions for antibiotics. Furthermore, it remained unclear how patients/prescriptions were selected, especially before the audit. The study included a low number of prescriptions (N = 55) which led to imprecise estimates and wide confidence intervals. However, compared to other studies, the risk of selective reporting was low as the internal computer software was used to obtain the data on prescriptions.

The external validity of the study by Chopra et al.[70] was similar to the study conducted by Zahabiyoun and colleagues.[69] As most patients of the participating department required emergency treatment, the external validity of the study might be low. In particular, it was unclear whether there were any differences between patients included before the audit and those investigated after the audit, for example in demographic variables such as age and sex.

In Raunair et al.[64] the risk of selection bias was high as only patients of dentists participating in the intervention were included. This study failed to state how the baseline data were collected from participating dentists and the authors did not explain how the data collected on prescriptions related to dentists. In particular, no information linking the number of prescriptions to the number of dentists and the numerical development of participating dentists over time (attrition) could be found. Furthermore, although authors collected demographic data, this data was not provided for each point of measurement, therefore it was not possible to assess differences across groups.

Finally, in Thomas and Hill[71] unknown risk of bias arose from the fact that a lot of information on data collection procedures and data analyses was not provided in their publication. It remains unclear how the information was collected (electronically or by hand), by whom (dental surgeon, nurse or someone else) and whether data collection was standardised or not. These factors offer the potential for a high risk of selective reporting and informational bias.

### Risk of bias across studies

As with all studies that focus on a change in behaviour, it was theoretically possible for the Hawthorne effect to occur in all included studies. This effect might have been augmented in several studies as dentists were asked to provide information every time they prescribe an antibiotic.[65–67] We viewed this procedure as problematic when considering selective reporting. Only few studies made use of objective data collection procedures: Elouafkaoui et al.[63] used NHS treatment claim data, Zahabiyoun et al.[69] made use of computer records and Chopra et al.[70] relied on patient records. However, in this study, it was unclear whether data extraction from records alone provided sufficient relevant information for the data sheet.

Apart from the Scottish study by Elouafkaoui et al.[63], every study exclusively relied on the voluntary enrolment of interested dentists in order to select their participants. Consequently, selection bias might have occurred as dentists not interested in increasing the accuracy of their prescriptions for antibiotics (i.e. poor prescribers) would be unlikely to participate.

Especially in the audits[65, 66, 68], the roles of study participants, study personnel and study conductors were not clear-cut but highly interwoven. To be specific, outcome measures were known by participants in most studies. In Raunair et al.[64] investigators explicitly mentioned and discussed the WHO prescribing indicator method used as an outcome measure.

**Fig 5:**
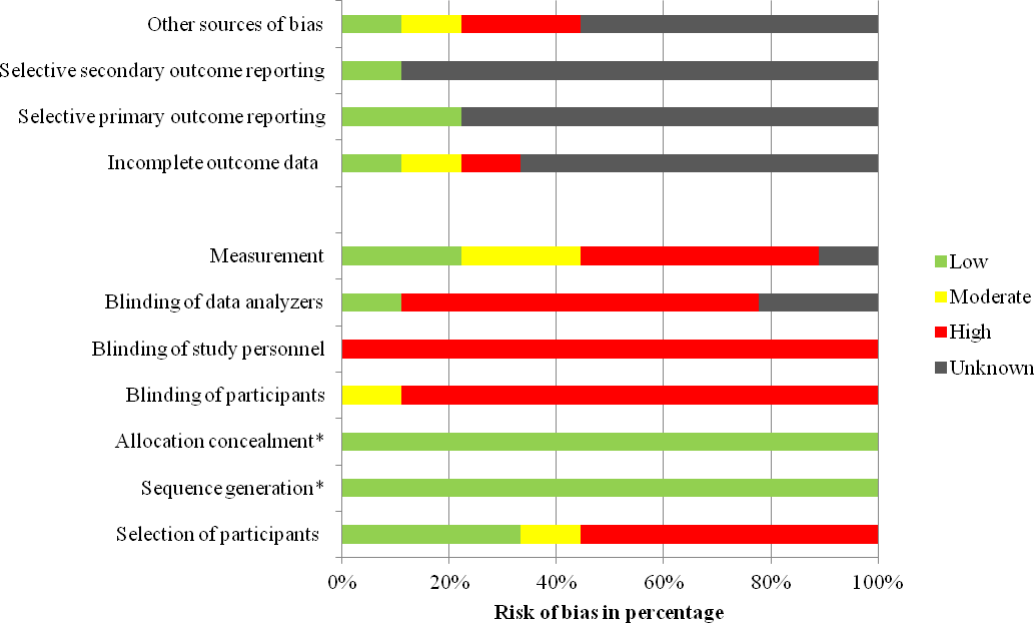
Risk of bias across included studies. Note: * Allocation concealment and sequence generation apply only to RCTs (Seager et al. and Elouafkaoui et al.) and are not applicable to pre-post studies. RCT = randomised controlled trial.

Furthermore, almost no study addressed incomplete outcome data due to missing data or participants leaving the study. For example, effects on the studies’ findings with respect to variance across groups largely remained unclear. In the study by Chate et al.[65], for instance, participants might have discontinued their participation during the 12-week audit period as this information was not available (personal correspondence). Basic demographic data pertaining to the participants was rarely collected and reported. Exceptions include Seager et al.[67], Thomas and Hill[71] and Raunair and colleagues.[64] However, the latter did not report this information over all time points. A different demographic composition of participants across groups and at different time points (e.g. with respects to age or sex) might explain differences in prescribing behaviour. Only Seager et al.[67] controlled for these variables in data analyses. A summary of the risk of bias across studies is provided in Fig 5. Last but not least, the risk of publication bias can be assumed to be low, as the search in Current Controlled Trials and ClinicalTrials.gov identified three ongoing trials[42, 62, 63] and did not yield any past studies with unpublished findings.

## Discussion

### Summary of evidence

Despite broad inclusion and exclusion criteria, only nine studies were identified that aimed to reduce the inadequate prescription of antibiotics in dentistry. Compared to general medicine and other medical fields, this number was considered to be extremely low. Five studies aimed to optimise the prescription of antibiotics in primary dental care[63, 65–68], while four other studies focused on outpatient dental care.[64, 69–71]

Interventions used in primary dental care mainly included a combination of audit, feedback, education, local consensus, dissemination of guidelines and/or academic detailing. On the other hand, trials in the outpatient setting made use of expert panel discussions, educational feedback on previous acts of prescribing, the dissemination of guidelines and the establishment of internal guidelines.

All studies were successful in reducing the number of antibiotics prescribed and in increasing the accuracy of the prescription. The strongest effects were found among studies conducted in the outpatient setting.[70, 71] However, these studies suffered from a low number of patients. Furthermore, these studies might lack external validity as departments with a high rate of prescribing antibiotics were the focus of these trials. Studies conducted in primary dental care contrasted with those administered in outpatient settings as the number of patients were usually higher. These trials reached lower but still remarkable levels of reductions in the prescription of antibiotics[65–67] and the adherence to prescribing guidelines for antibiotics increased.[65]

However, the findings should be treated with caution due to the poor study reporting and high risk of bias: Firstly, several publications provided only limited information on design and conduct of the respective study[64, 68, 70, 71] and the risk of bias within these studies could not be resolved. Secondly, most studies suffered from a high risk of selection bias as participation had been based on free will, rather than a standardised sampling procedure.[65, 66, 68] Frequently, the participating dentists and clinics were selected specifically because of their high prescribing behaviour.[69, 70] Thirdly, in several studies participants had been critically involved in the study design, conduct and analyses[58, 65, 68], giving rise to a number of different sources of bias. Furthermore, data collection procedures were largely based on regular voluntary reporting by dentists[58, 65, 67], so that the risk of selective reporting on the prescription of antibiotics was high. Moreover, variances between study groups could not be ruled out, as there was a lack of information regarding participants and clinical conditions. Last but not least, only three studies related the number of prescriptions for antibiotics to the number of patients in treatment.[63, 67, 71] Fluctuations in the number of treated patients will inevitably have an impact on the number of prescriptions when not appropriately considered.

Ultimately, several of these limitations might have been strongly related to the fact that some studies were conducted as audits rather than as scientific studies.[58, 65, 68] Because of this, the authors were less inclined to take heed of the scientific standards, including sample size calculations, publication of a study protocol or adherence to study procedures such as blinding and objective data collection.

### Strengths and limitations

A major strength of this systematic review was its very broad search frame: In order to identify studies aiming to optimise the prescription of antibiotics in dentistry, publications published since 1960 were searched without a geographical restriction. All study designs such as general and specialised dentistry and a combination of both were included. Publication bias was reduced by extending the search to two large databases concerned with the registration of randomised controlled trials.

This systematic review could offer conclusions which took the limitations of the investigated studies into account. Unfortunately, few studies could be included and many of these studies were confounded by a low quality of scientific reporting and lack of information regarding design and conduct of the studies. Only two RCTs could be included in this review, while all the other studies had not been randomised, giving rise to a number of potential biases. These included selective reporting of outcomes, reporting bias and limited capacity to ascertain causality.[54] In fact, only the RCTs[63, 67] had a published protocol. Additionally, reporting of outcome measures was limited. Confidence intervals, p-values and standard deviations were almost never reported, so that a meta-analysis could not be conducted based on the provided data. The number of antibiotics prescribed within the trials were measured in different ways, which might have an impact on comparisons between studies. Finally, only one study addressed adverse effects owing to the reduction of prescriptions for antibiotic drugs [71] and most studies had comparatively short follow-up periods. Assessing long-term effects of interventions was hardly possible.

### Findings in light of existing research

All interventions included in this systematic review were able to reduce the number of prescriptions for antibiotics and to critically enhance the adherence to guidelines. This corroborated findings from other medical fields.[75, 76] Focusing on primary care, Arnold and Straus compiled a Cochrane systematic review in 2005. They found that multi-faceted interventions including educational elements, aimed at patients and physicians alike, were most successful in reducing the number of prescriptions for antibiotics. In order to be efficient, interventions should address local barriers. Most interestingly, the effect size, which was necessary to reduce the incidence of antibiotic-resistant bacteria was taken into account by both authors.[76] In 2014, Roque et al., who focussed on primary and secondary care, confirmed these findings, but disregarded the necessary effect size to reduce antibiotic resistance.[75] Both reviews provided evidence that passive interventions such as the dissemination of guidelines alone are less effective than active interventions including discussion, educational meetings or individual feedback on acts of prescribing.[75, 76] Seager et al. were the first to provide evidence that this is also true within dentistry.[67]

Moreover, the existence of local barriers to changing established practice was addressed by individual studies. As an example, Chate et al. identified pain, patient expectations, time pressure, workload, the uncertainty of the diagnosis and a patient holiday as factors related to inappropriate acts of prescribing antibiotics.[65] Chopra et al. mentioned time pressure and patient expectations.[70] These findings complemented the factors found in qualitative research and highlighted the dentists’ desire to avoid clinical complications, the fear to lose patients and awareness of patient pressure.[41, 42] Obviously, factors impacting on inadequate acts of prescribing might be different from one setting to the other and might change over time. Zahabiyoun et al. have emphasised correctly, that 100% compliance with guidelines can only be achieved by addressing all underlying reasons for inappropriate acts of prescribing antibiotics.[69] Additionally, some studies emphasised that more cycles of audits are necessary to further improve prescribing.[65, 66, 69]

Unfortunately, there was too little evidence for this review to assess which interventions work best in which setting. However, dental outpatient departments were more likely to employ expert panel discussions, educational feedback on previous acts of prescribing, the dissemination of guidelines and the establishment of internal guidelines compared to other interventions.[64, 69–71] Interventions in general dental care used a combination of audit, feedback, education, local consensus, dissemination of guidelines and/or academic detailing.[63, 65–67] Interventions in both settings might have aimed to satisfy specific requirements. Furthermore, it seemed plausible that improvements in acts of prescribing antibiotics were more salient in departments (as dentists work under supervision) than in general dentistry (where the organisational structures are flatter and dentists might even be autonomous). The higher degree of collaboration between the dentist and the researcher in smaller compared to larger studies might also explain these differences. These interventions might be characterised as active and frequent which would render these more efficient.

Finally, to oppose antibiotic resistance development, it has become necessary to mobilise initiatives in many fields that are able to address different conditions, settings and circumstances all over the world. In fact, the WHO action plan focused not only on optimising the use of antibiotics but also on awareness and understanding of resistance development, surveillance, infection prevention and on novel medicines, diagnostic tools and vaccines.[8] The realistic assessment of the effects these initiatives might have on antibiotic resistance development is a necessary but often disregarded step. However, it is worthwhile: Sweden was able to steadily decrease the use of antibiotics between 1990 and 2004 by following the Swedish Strategic Programme Against Antibiotic Resistance. The programme was based on a network of local multidisciplinary groups providing prescribers with feedback and a national executive working group coordinates activities.[77]

On the other hand, adverse effects owing to the reduction of the number of prescriptions for antibiotics have been rarely assessed. In a recent systematic review Cahill et al.[78] investigated the evidence for use of antibiotic prophylaxis for prevention of bacteraemia or infective endocarditis in patients undergoing dental procedures. They concluded that the evidence base was limited, heterogeneous and the methodological quality of many studies was not up to standard. Consequently, this question still remains controversial and requires further research.

### Generalizations

Despite high levels of global misuse and overuse of antibiotics in dentistry, this systematic review provided evidence for the very low number of studies addressing the improvement of appropriate acts of prescribing antibiotics in this field. Studies included from both general dental care and outpatient care showed that interventions were successful in reducing the number of prescriptions for antibiotics and in increasing adherence to local guidelines. However, given the lack of high-quality study design/methodology, studies were susceptible to different types of bias with selection bias and selective outcome reporting being the most prevalent. Notwithstanding, the existing evidence gave reason to assume that interventions used in these studies were able to optimise acts of prescribing antibiotics in dental care to a considerable extent.

### Future research

To assess the effect of interventions aiming to improve acts of prescribing antibiotics in dentistry, future research should focus on the design and methodology of high-quality RCTs. Objective outcome reporting measures (such as software-based reporting) and standardised outcome measures (e.g. relating number of antibiotics prescribed to number of patients or treatments) should be employed. Furthermore, studies should aim for longer periods of follow-up (such as one or two years), rigorous methodology (such as blinding) and adequate standard of study reporting (including the publication of study protocols). Future studies may also focus on the effects of interventions on adverse outcomes, costs and antibiotic resistance development. In the presence of high-quality evidence alone, it will eventually become possible to assess which type of intervention is most effective in which setting.

## Supporting information

S1 PRISMA Checklist (DOC)

## Abbreviations

CI: Confidence Interval
DDD: Defined Daily Dose
GDP: General Dental Practitioner
NICE: UK National Institute for Health and Clinical Excellence
NHS: National Health Service
RCT: Randomised Controlled Trial
WHO: World Health Organization
